# DIGITAL AGEISM AND ITS IMPLICATIONS FOR OLDER ADULT INCLUSION

**DOI:** 10.1093/geroni/igad104.1036

**Published:** 2023-12-21

**Authors:** Charlene Chu

**Affiliations:** University of Toronto, Toronto, Ontario, Canada

## Abstract

“Digital ageism” is a new form of ageism that is embedded into technology and AI systems. Building on the World Health Organization’s recently published policy brief entitled “Ageism in AI for Health”, our work draws attention to digital ageism referring to the nexus of ageism (discrimination or bias related to age) that is mediated and perpetuated by artificial intelligent (AI) systems and technologies. This paper presents a conceptual framework for identifying various cycles of injustice that perpetuate ageism into our digital spheres. This framework also identifies key contributors that need to be addressed in order to break these cycles. We present the results of a scoping literature review, informed by Arksey and O’Malley, to identify the mechanisms in which digital ageism can be introduced as demonstrated in the literature. This work contributes to foundational knowledge about age-related biases in AI and how they might be encoded or amplified in AI systems. This work sheds light on the need for ethical thinking and other approaches to better include older people, and ensure technology benefits the aging population.

